# Endurance Exercise Enhances Emotional Valence and Emotion Regulation

**DOI:** 10.3389/fnhum.2018.00398

**Published:** 2018-10-16

**Authors:** Grace E. Giles, Marianna D. Eddy, Tad T. Brunyé, Heather L. Urry, Harry L. Graber, Randall L. Barbour, Caroline R. Mahoney, Holly A. Taylor, Robin B. Kanarek

**Affiliations:** ^1^Department of Psychology, Tufts University, Medford, MA, United States; ^2^Cognitive Science Team, US Army Natick Soldier, Research, Development, and Engineering Center (NSRDEC), Natick, MA, United States; ^3^Center for Applied Brain and Cognitive Sciences, Tufts University, Medford, MA, United States; ^4^SUNY Downstate Medical Center, Brooklyn, NY, United States

**Keywords:** exercise, cognitive control, emotion regulation, cognitive reappraisal, prefrontal cortex (PFC), functional near infrared spectroscopy (fNIRS)

## Abstract

Acute exercise consistently benefits both emotion and cognition, particularly cognitive control. We evaluated acute endurance exercise influences on emotion, domain-general cognitive control and the cognitive control of emotion, specifically cognitive reappraisal. Thirty-six endurance runners, defined as running at least 30 miles per week with one weekly run of at least 9 miles (21 female, age 18–30 years) participated. In a repeated measures design, participants walked at 57% age-adjusted maximum heart rate (HR_max_; range 51%–63%) and ran at 70% HR_max_ (range 64%–76%) for 90 min on two separate days. Participants completed measures of emotional state and the Stroop test of domain-general cognitive control before, every 30 min during and 30 min after exercise. Participants also completed a cognitive reappraisal task (CRT) after exercise. Functional near-infrared spectroscopy (fNIRS) tracked changes in oxygenated and deoxygenated hemoglobin (O_2_Hb and dHb) levels in the prefrontal cortex (PFC). Results suggest that even at relatively moderate intensities, endurance athletes benefit emotionally from running both during and after exercise and task-related PFC oxygenation reductions do not appear to hinder prefrontal-dependent cognitive control.

## Introduction

Aerobic exercise is thought to influence emotion and cognitive control in a dose-dependent manner (Dietrich, 2006; Ekkekakis and Acevedo, 2006). That is, exercise intensity generally determines the magnitude and direction of exercise effects on emotion and cognitive control. Cognitive control involves selecting and responding to goal-relevant stimuli while inhibiting attention toward conflicting information (Miyake et al., 2000). According to the “dual-mode” theory, at low to moderate intensities, cognitive factors, such as exercise self-efficacy and appraisals of goals, yield positive emotional responses, whereas at higher intensities, interoceptive factors, such as muscular and cardiovascular cues, yield negative emotional responses (Ekkekakis and Petruzzello, 1999; Ekkekakis, 2003). Likewise, according to the “reticular-activating hypofrontality (RAH)” model, increasing exercise intensity shifts the balance in cerebral metabolic resource allocation from structures supporting cognitive control, namely the prefrontal cortex (PFC), to those supporting body movement, including motor, sensory and autonomic pathways (Dietrich, 2003, 2006; Dietrich and Audiffren, 2011). Studies examining short duration, moderate-intensity exercise effects on cognitive control generally report beneficial effects (Chang et al., 2012; Lucas et al., 2012; Basso and Suzuki, 2017). Exercise for longer durations, termed endurance exercise, is becoming increasing popular (Douglas and Fuehrer, 2014). However, research on endurance exercise effects on emotion and cognitive control remains relatively limited.

Among the few studies to have evaluated endurance exercise effects on emotion and domain-general cognitive control, endurance exercise ranging from a 1 h to a 6-day run benefited multiple aspects of emotion, including lowering tension, anger and depression and elevating happiness and calmness (Markoff et al., 1982; Lane and Wilson, 2011; Parry et al., 2011). A 1 h run also impaired cognitive control domains including working memory, perseveration, set shifting and sustained attention (Dietrich and Sparling, 2004), however other research suggests that decrements in attentional control do not begin until the second hour of exercise (Grego et al., 2004). Such results suggest that endurance exercise generally enhances emotion and may either enhance or impair cognitive control, at least among individuals who likely have trained for such events and are physically fit. However, extant research focuses on changes in emotion from before to after endurance exercise and does not address fluctuations that may occur over the course of it.

Neuroimaging may help to clarify exercise-dependent changes in frontal regions of the brain implicated in cognitive control. Functional near-infrared spectroscopy (fNIRS) measures changes in oxygenated and deoxygenated hemoglobin (O_2_Hb and dHb) from superficial layers of cortex (Cui et al., 2011). Thus, in comparison to functional magnetic resonance imaging, a smaller portion of the brain is accessible to fNIRS measurements. However, advantages afforded by the use of fNIRS for exercise-based studies include near-complete freedom from movement restrictions and high temporal resolution (Piper et al., 2014). Also, while the portability of electroencephalography is comparable to that of fNIRS, the latter has the advantage of lower susceptibility to muscle-contraction and motion artifacts (Balardin et al., 2017). Prior fNIRS-based studies have shown that increased O_2_Hb and decreased dHb generally indicate regional cortical activation (Villringer et al., 1993). Total hemoglobin (tHb) is the sum of O_2_Hb and dHb. Changes to dHb are generally smaller than those to O_2_Hb, and thus changes to O_2_Hb and tHb are often similar (Ehlis et al., 2005). At relatively low to moderate intensities, exercise increases O_2_Hb in the PFC, whereas at high intensities, when exercise reaches or exceeds maximum oxygen uptake (VO_2max_, a measure of cardiorespiratory fitness), O_2_Hb often declines, though to a lesser extent in trained than untrained individuals (Rooks et al., 2010).

Given the evidence that exercise influences domain-general cognitive control, as well as associated changes in PFC oxygenation, it follows that exercise may influence more specific aspects of cognitive control, such as the cognitive control of emotion, i.e., emotion regulation. Emotion regulation refers to cognitive processes that enable individuals to regulate their own emotions, through both conscious and non-conscious processes, by increasing or decreasing the experience of negative or positive emotions (Gross, 1999). Emotion regulation and emotion induction paradigms have been shown to both increase and decrease PFC O_2_Hb and tHb (Herrmann et al., 2003; Leon-Carrion et al., 2006; Glotzbach et al., 2011; Giles et al., 2017). Self-report studies suggest that exercise ranks first among methods deemed successful in changing negative emotions (Thayer et al., 1994) and that regular exercisers valued the emotion improving effects of exercise more so than individuals just beginning an exercise regimen (Hsiao and Thayer, 1998). However, no study has experimentally manipulated how exercise may influence individuals’ abilities to regulate their emotions in response to emotional situations. Of particular interest is the emotion regulation strategy known as cognitive reappraisal, which involves reevaluating emotional stimuli in order to augment or reduce their emotional impact (Gross, 2002), as it is generally successful in enhancing positive and reducing negative emotion experience (Webb et al., 2012). Recent research suggests that regular exercise is associated with enhanced cognitive reappraisal success (Giles et al., 2017), but no study has evaluated whether acute exercise has similar effects.

Given the increasing popularity of endurance running events (Douglas and Fuehrer, 2014), and little consensus as to how running such distances influences emotion and cognitive control, the present experiment aimed to determine whether endurance exercise influences domain-general cognitive control, the cognitive control of emotion, and associated changes in PFC oxygenation. The approach taken was to have experienced runners exercise on a treadmill, at two different levels of exercise intensity (i.e., walking and running), and to evaluate their emotional states and cognitive control at five points in each session (one before, three during, and one after exercise). In addition, fNIRS measurements over the PFC were collected throughout each session.

Domain-general cognitive control was measured via the Stroop test of selective attention and response inhibition (Stroop, 1935; Botvinick et al., 2001; but see also, Soutschek and Schubert, 2013). Cognitive control of emotion was measured via the cognitive reappraisal task (CRT; Urry, 2009). Based on previous evidence that short-duration, moderate-intensity exercise enhances and longer-duration exercise impairs cognitive control and PFC oxygenation (Dietrich and Sparling, 2004; Rooks et al., 2010; Chang et al., 2012; Giles et al., 2018), we predicted that response inhibition on the Stroop test and O_2_Hb in the PFC would increase in the first hour of the Run relative to the Walk, then decrease in the second hour. Based on evidence that cognition improves after exercise (Chang et al., 2012), we predicted that cognitive reappraisal success would be greater following the Run than Walk.

A secondary objective was to assess whether endurance exercise influences emotion. Given that acute exercise tends to enhance positive emotion in physically fit individuals (Reed and Ones, 2006; Ekkekakis et al., 2011), we hypothesized that positive emotion and arousal would be higher during the run than during the walk.

## Materials and Methods

### Participants

Thirty-six individuals (21 female, 15 male; age 18–30 years) participated for monetary compensation of $150 USD (see Table 1). All participants were right-handed with normal color vision. Participants were required to regularly run at least 30 miles per week, with at least one run of 9 miles or more. This minimum was chosen because participants were expected to run approximately 9 miles within the 90-min study, given that the median half marathon pace is the United States is approximately 10 min per mile (Running USA, 2014). Participants were required to have experience with and be comfortable running on a treadmill. Participants did not take anxiolytic and/or antidepressant medications and had no history of seizure, head injury (including neurosurgery and concussions), illness that caused brain injury, any other brain-related condition (such as traumatic brain injury), history of a neurological or psychological disorder, metal in the head, or a sensitive scalp. The protocol was approved by the Tufts University Institutional Review Board and the Army Human Research Protections Office. All participants gave written informed consent in accordance with the Declaration of Helsinki.

**Table 1:** Sample characteristics (*n* = 36).

	Average	SD	Minimum	Maximum
Age	23.4	3.6	18	30
Weekly running total distance	36.9	9.3	30	65
Miles (kilometers)	(59.4)	(15.0)	(48.3)	(104.6)
Weekly long run	11.8	3.3	9	26
Miles (kilometers)	(19.0)	(5.3)	(14.5)	(41.8)
BMI	22.2	3.6	17.6	38.2
Godin total leisure time	80.5	24.6	42	125
Beck depression inventory	2.2	2.3	0	9
Perceived stress scale	17.0	6.4	6	37
State-trait anxiety inventory—trait	33.6	5.3	23	50
**Emotion regulation Questionnaire**
Reappraisal	5.2	0.7	4.0	6.8
Suppression	3.5	1.3	1.0	5.8

### Design

The experiment used a repeated measures design, with Exercise Intensity (Walk, Run) as the within-participants factor. Sample size estimation was based on effect sizes from Ando et al. (2011), who found that exercise at 80% VO_2 max_ increased error rate during the Eriksen flanker task of response inhibition (*η^2^* = 0.21). Using GPower (Faul et al., 2007), the necessary sample size was estimated to be 15 with an alpha level of *p* = 0.05 and a power of 0.95, using repeated measures analysis of variance (ANOVA) with one degree of freedom.

### Questionnaires

The Godin Leisure Time Questionnaire (Godin and Shephard, 1985), Beck Depression Inventory (BDI; Beck et al., 1961), Perceived Stress Scale (PSS; Cohen et al., 1983), Emotion Regulation Questionnaire (Gross and John, 2003), and State-Trait Anxiety Inventory—Trait (Spielberger et al., 1983) were administered to capture sample characteristics. For complete scale descriptions and internal consistency assessments (α coefficients), see Supplementary Appendix A. Indeed, for depression, all participants fell within the cut-off for “minimal depression” (Beck et al., 1988). For anxiety, only one participant met the cut-off of 46 for as the delineation between functional and clinical regions of the distribution (Fisher and Durham, 1999). The same participant scored high for perceived stress (see Table 2 for distribution of depression, anxiety and stress scores). Exploratory removal of this participant did not change the reported patterns.

**Table 2:** Distribution of Beck Depression Inventory (BDI), State-Trait Anxiety Inventory -Trait (STAI-Trait) and Perceived Stress Scale (PSS) scores (*n* = 36).

	<10	10–18	19–29	30–63
BDI	*n* = 36	*n* = 0	*n* = 0	*n* = 0
	20–30	30–40	40–50	>51
STAI	*n* = 9	*n* = 23	*n* = 4	*n* = 0
	0–10	11–20	21–30	31–40
PSS	*n* = 2	*n* = 26	*n* = 7	*n* = 1

#### Feeling Scale (FS)

The feeling scale (FS) is a one-item inventory measuring the extent to which participants feel pleasant or unpleasant and ranges from “very good” (+5) to “very bad” (−5; Hardy and Rejeski, 1989).

#### Felt Arousal Scale (FAS)

The felt arousal scale (FAS) is a one-item inventory measuring feelings of arousal and ranges from “low arousal” (1) to “high arousal” (6; Svebak and Murgatroyd, 1985).

#### Borg Rating of Perceived Exertion (RPE) Scale

The rating of perceived exertion (RPE) is a commonly used one-item self-report measure of perceived physical exertion and ranges from “no exertion at all” (6) to “maximal exertion” (20; Borg, 1982). For RPE and heart rate (HR) results, see Supplementary Appendix B.

### Domain-General Cognitive Control: Stroop Test

The Stroop test (Stroop, 1935) is a classic test of selective attention and response inhibition, in which participants are shown words (i.e., *red*, *green*, *yellow* and *blue*) that appear in a font color (i.e., red, green, yellow or blue) that is either congruent (e.g., *red* appearing in red ink) or incongruent (e.g., *red* appearing in blue ink) with the meaning of the word. Participants were asked to respond to the ink color without reading the word by pressing the relevant colored keyboard key. Four key orders, one key for each ink color, were generated, such that each color appeared in each of the four key positions once. Key orders were constant within participants and counterbalanced across participants, such that an equal number of participants were given each key order. Trials were presented in pseudo-random order, such that no two words or colors (i.e., red, blue, green, yellow) or conditions (i.e., congruent, incongruent) repeated more than twice in a row. The task included 48 trials (24 congruent, 24 incongruent) with 2 s maximum response time and a 9–13.5 s variable inter-stimulus interval. A 9–13.5 s variable inter-stimulus interval was chosen based on studies assessing the influence of exercise on Stroop-related PFC oxygenation (Hyodo et al., 2012; Byun et al., 2014) to ensure that the hemodynamic response returned to baseline between trials, which occurs within 8–10 s (Huppert et al., 2013).

Dependent measures included accuracy and response time (on accurate trials only). “Stroop interference” was calculated by the difference in response time between incongruent and congruent trials, with lower scores reflecting enhanced performance.

### Cognitive Control of Emotion: Cognitive Reappraisal Task (CRT)

The CRT involves viewing a series of negative pictures from the International Affective Picture System (IAPS; Lang et al., 2005) while attempting to either reappraise, i.e., imagine the situation in the picture improving; or maintain thoughts of the pictures, i.e., imagine the situation in the picture staying the same (Urry, 2009). In each trial, participants first viewed the picture (4 s; “preparatory period”), then heard an auditory instruction to decrease or maintain. They then continued to view the picture during which time they were intended to employ their instructed strategy (8 s; “regulation period”). Participants then rated the unpleasantness of the picture on a seven-point scale ranging from “not at all” (1) to “very” (7). For a schematic of trial structure, see Supplementary Appendix C. Reappraisal success was quantified by the difference between Negative/Maintain and Negative/Reappraise (i.e., Negative/Maintain—Negative/Reappraise) ratings of unpleasantness, with higher scores indicating more successful reappraisal. A total of 72 trials were presented, including 24 trials of neutral pictures (normative valence ratings mean ± SD = 5.3 ± 0.7, arousal = 4.0 ± 0.8 on the Self-Assessment Manikin (SAM) ranging from low pleasure or arousal (1) to high pleasure or arousal (9; Lang and Bradley, 1997)) with the instruction to maintain and 48 trials of unpleasant pictures (normative valence = 2.2 ± 0.5, arousal = 5.9 ± 0.8), half with the instruction to maintain and half with the instruction to decrease. The negative pictures were randomly assigned to the two instructions for each participant.

Participants completed an open-ended response questionnaire, including the two items, “What strategies did you use to maintain?” and “What strategies did you use to decrease?” Open-ended responses were coded according to Opitz et al. (2015): whether participants utilized: (1) cognitive reappraisal; (2) other emotion regulation strategies such as attentional deployment, response modulation, or imagining the pictures as in some way not real; and (3) more than one strategy. All three categories were coded as 1 = Yes and 0 = No, such that the means indicate percentage of participants whose responses indicated their use of the strategy(s) in question. Coding responses from two investigators were compared, and only matching codes were submitted to subsequent analyses. Inter-rater reliability was evaluated by computing Randolph’s free-marginal multirater kappas (Randolph, 2005, 2008) and resulted in adequate agreement among investigators (all *κ* > 0.75).

### Functional Near-Infrared Spectroscopy (fNIRS)

**Figure 1:**
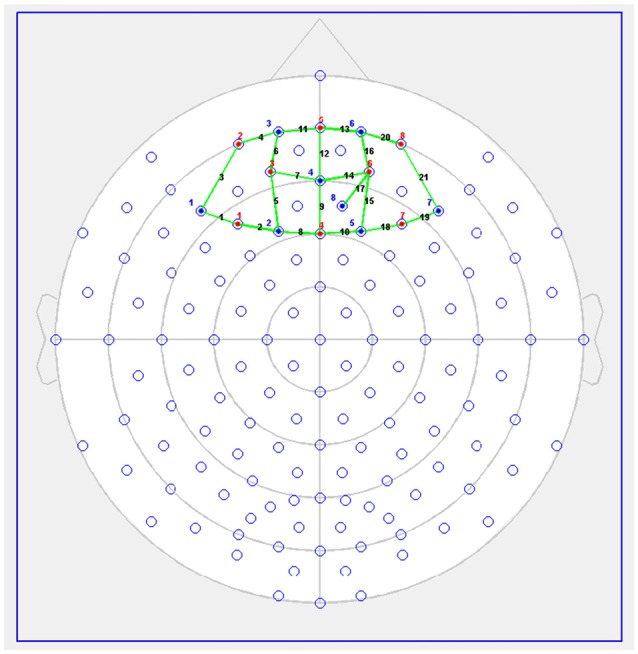
Functional near-infrared spectroscopy (fNIRS) Probe Setup (red dots = sources, blue = detectors). Figure previously published in Giles et al. (2017; copyright license number 4436570673169).

fNIRS was performed using the NIRSport (NIRx Medical Technologies, LLC, Glen Head, NY, USA). This continuous-wave fNIRS system consisted of eight light sources and eight detectors, with a 3 cm source-detector separation comprising 21 channels, all across the dorsal and anterior PFC (see Figure 1). Each LED light source emitted light at two wavelengths (760 and 850 nm). Data were recorded at 7.81 Hz.

### Procedure

Participants completed three sessions: one practice session and two experimental sessions. During the practice session, interested participants first completed the consent and were screened for eligibility. If participants met inclusion and exclusion criteria described above, they completed the Godin Leisure Time Questionnaire, BDI, PSS, Emotion Regulation Questionnaire, and trait subscale of the State-Trait Anxiety Inventory. They then completed practice trials of the Stroop test while walking and running.

**Figure 2:**
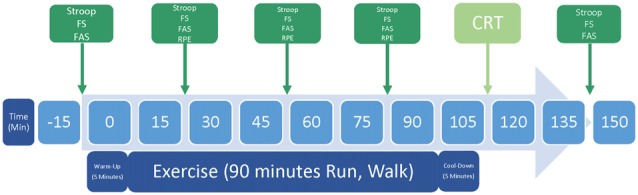
Schematic representation of one study session. The experiment consisted of two experimental sessions, during which participants either Walked or Ran for 90 min. Abbreviations include the feeling scale (FS), felt arousal scale (FAS), rated perceived exertion scale (RPE) and cognitive reappraisal task (CRT).

During each of the two experimental sessions, participants first donned the fNIRS and HR monitors (Polar model RS800CX) and completed a baseline set of FS and FAS (verbal report), and Stroop test (keyboard press). They warmed up by walking for 5 min at 2.5 miles per hour (MPH), i.e., 4.0 kilometers per hour (KPH). Participants then walked at 57% age-adjusted maximum HR (HRmax; range 51%–63%) or incrementally increased the speed until running at 70% HRmax (range 64%–76%), after which they adjusted their speeds to remain with the prescribed HR zone, for 90 min. The Walk and Run conditions were completed in counterbalanced order. They were chosen based on the American College of Sports Medicine’s classification of exercise intensity as light and moderate, respectively (Garber et al., 2011). During the Walk and Run, participants completed the RPE, FS, FAS, and Stroop test every 30 min. Participants then cooled down by walking for 5 min at 2.5 MPH (4.0 KPH). Upon completion of exercise and cool-down, participants took a seat to continue recovery. After 5 min, participants completed the CRT. fNIRS was recorded during each Stroop test and the CRT. Finally, the participants completed the last FS, FAS and Stroop test. See Figure 2 for schematic of exercise sessions.

Exercise sessions were separated by at least 1 week to minimize injury risk and practice effects on the tasks. To reduce diurnal variation in cognitive and physical performance, within-participant test sessions were scheduled for approximately the same time of day (± 1 h). To reduce the influence of hydration status on cognitive and physical performance, participants were asked to consume $\frac{1}{2}$ liter of water the night before a test session, and $\frac{1}{2}$ liter of water the morning of a test session. Participants were also required to consume at least one meal prior to a morning test session (i.e., breakfast), and at least two meals prior to an afternoon test session (i.e., breakfast, lunch). They were asked to abstain from moderate to heavy exercise, caffeine, alcohol and dietary supplement intake for 24 h prior to the experiment. Compliance to instructions was confirmed via a self-report checklist. The two experimental sessions were identical with the exception of the exercise intensity. Following the second experimental session, participants were fully debriefed and compensated for their participation.

### Statistical Methods

#### Behavioral Data

Stroop test response time and accuracy were analyzed using repeated measures ANOVA with Intensity (Run, Walk), Time (Pre-Exercise, Minute 30, Minute 60, Minute 90, Post-Exercise) and Congruency (Congruent, Incongruent) as fixed factors. For Stroop results analyzed separately for before vs. after exercise and within exercise, see Supplementary Appendix D. CRT unpleasantness ratings were first analyzed using repeated measures ANOVAs with Intensity (Run, Walk) and Picture Valence (Negative/Maintain, Neutral/Maintain) as fixed factors to test whether the pictures induced negative emotion as intended, and second, with Intensity (Run, Walk) and Instruction (Negative/Maintain, Negative/Decrease) as fixed factors to test whether the sample exhibited successful reappraisal. Reappraisal success was calculated as by the difference between Negative/Reappraise and Negative/Maintain (i.e., Negative/Maintain − Negative/Reappraise) ratings of unpleasantness, with higher scores indicating more successful reappraisal. Reappraisal success scores following the Run and Walk were submitted to paired samples *t*-tests. Coded values from the open-ended questionnaire were submitted to single-sample *t*-tests with test values of 0.

FS, FAS and RPE data were analyzed using the aligned rank transform (ART) for nonparametric factorial data analysis (Wobbrock et al., 2011). Repeated-measures ANOVAs were then performed on the transformed data, with Intensity (Run, Walk) and time (Pre-Exercise, Minute 30, Minute 60, Minute 90, Post-Exercise) as fixed factors. To evaluate any exercise Intensity by Time interactions, pairwise comparisons were conducted using Wilcoxon signed-rank tests on the original data. Effect sizes are presented as eta-squared for ANOVA and Cohen’s *d* for *t*-tests.

An effect was deemed statistically significant if the probability of its occurrence by chance was *p* < 0.05. When sphericity was violated, Greenhouse–Geisser corrected *p*-values were used. When an ANOVA yielded a significant interaction effect, *post hoc* tests using the Bonferroni correction were conducted. All statistical analyses described above were performed using SPSS 12.0.

#### fNIRS Data

The NIRStar acquisition software (NIRx Medical Technologies, LLC, Glen Head, NY, USA) was used to record fNIRS data and to evaluate its signal-to-noise ratio. The nirsLAB data analysis package (NIRx Medical Technologies, LLC, Glen Head, NY, USA) was used for all subsequent calculations. Raw data for all channels were visually inspected, spike artifacts were removed, and faulty channels were removed from subsequent analyses (an average ± SD of 1.24 ± 1.85 channels per task iteration for the Stroop test, and 0.21 ± 0.63 for the CRT). All channels were band-pass filtered, with low cutoff frequency = 0.01 Hz and high cutoff frequency = 0.1 Hz. The modified Beer–Lambert law was used to compute estimates of changes in O_2_Hb, dHb and tHb (i.e., O_2_Hb + dHb) levels from the frequency-filtered data, using the 30–60 s time period before each task iteration as the baseline (Sassaroli and Fantini, 2004).

The Statistical Parametric Mapping (SPM) utilities incorporated into nirsLAB were used to determine event-related changes in O_2_Hb, dHb and tHb during the Stroop and CRT. SPM employs the general linear model (GLM) to identify O_2_Hb, dHb and tHb hemodynamic brain responses with reference to experimental factors. Level-1 analyses (SPM 1) assess differences on a within-participant basis and were used to generate parameter estimates (β weights) for each channel and factor, i.e., exercise Intensity, Time and Congruency for the Stroop test and exercise Intensity, picture Valence and regulation Instruction for the CRT.

Stroop test O_2_Hb, dHb and tHb β weights for each channel were analyzed using repeated measures ANOVAs with exercise Intensity (Run, Walk), Time (Pre-Exercise, Minute 30, Minute 60, Minute 90, Post-Exercise) and Congruency (Congruent, Incongruent) as within-participants factors. Similarly, cognitive reappraisal test β weights for each channel were analyzed using repeated measures ANOVAs with exercise Intensity (Run, Walk) and: (1) preparatory period: picture Valence (Negative, Neutral); or (2) regulation period: picture Valence/Instruction (Negative/Decrease, Negative/Maintain, Neutral/Maintain) as within-participants factors. Alpha levels were Bonferroni corrected for multiple comparisons (α = 0.05 divided by 21 channels resulted in α = 0.0024) (Kopton and Kenning, 2014; Piper et al., 2014).

## Results

### Preliminary Analyses of Emotional and Physiological Responses to Endurance Exercise

#### Feeling Scale (FS)

Emotional valence was more positive in the Run than in the Walk condition, *F*_(1,35)_ = 5.305, *p* = 0.027, *η*^2^ = 0.025. Emotional valence was more positive before and 30, 60 and 90 min into exercise than after exercise, *F*_(4,140)_ = 8.446, *p* < 0.001, *η*^2^ = 0.109 (see Table 3). No Intensity by Time Interaction was found (*p* = 0.541).

**Table 3:** Feeling scale (FS) and felt arousal scale (FAS) means, standard error of the means (SEM), medians, and interquartile ranges (ICQ) for each Exercise and Time (*n* = 36).

		Feeling scale				Felt arousal scale			
		Mean	SEM	Median	IQR	Mean	SEM	Median	IQR
10 Min	Run	2.7	0.3	3	3	1.8	0.1	2	1
Pre-exercise	Walk	2.4	0.3	3	3	1.8	0.2	1	2
Min 30	Run	3.1	0.3	3	2	2.8	0.1	3	1
	Walk	2.8	0.3	3	2	2.2	0.1	4	1
Min 60	Run	3.3	0.3	3	2	3.0	0.2	3	2
	Walk	2.7	0.3	3	1	2.4	0.2	2	1
Min 90	Run	3.4	0.4	3	2	3.3	0.2	3	1
	Walk	2.6	0.3	3	1	2.5	0.2	2	1
30 Min	Run	1.9	0.3	2	2	2.3	0.2	2	2
Post-exercise	Walk	1.6	0.3	2	3	2.0	0.2	2	2

#### Felt Arousal Scale (FAS)

Emotional arousal was higher in the Run than in the Walk condition, *F*_(1,35)_ = 16.491, *p* < 0.001, *η*^2^ = 0.103 (see Table 3). Further, emotional arousal was higher during 30, 60 and 90 min exercise than before and after exercise, *F*_(4,140)_ = 21.127, *p* < 0.001, *η*^2^ = 0.236. An Intensity by Time interaction, *F*_(4,140)_ = 6.481, *p* < 0.001, *η*^2^ = 0.050, showed that rated arousal was higher during the Run than the Walk during 30 min, *z* = 3.762, *p* < 0.001, 60 min, *z =* 2.514, *p* = 0.012 and 90 min, *z* = 3.299, *p* = 0.001, exercise, but not before or after exercise (all *p-*values > 0.12).

#### Borg Rating of Perceived Exertion (RPE) Scale

Perceived exertion was higher during the Run than Walk condition, *F*_(1,35)_ = 22.599, *p* < 0.001, *η*^2^ = 0.273 (mean ± SD Run = 12.22 ± 1.52, Walk = 10.15 ± 1.68). For full results, see Supplementary Appendix B.

#### Heart Rate

HR was higher during the Run than Walk condition, *F*_(1,34)_ = 390.001, *p* < 0.001, *η*^2^ = 0.720 (mean ± SD Run = 145.17 ± 13.82, Walk = 100.28 ± 8.79). For full results, see Supplementary Appendix B.

### Primary Analyses of Cognitive Control Responses to Endurance Exercise

#### Does Endurance Exercise Influence Domain-General Cognitive Control and Associated Changes in PFC Oxygenation on the Stroop Test?

##### Behavioral Results

Consistent with classic findings (Stroop, 1935), response times were faster, *F*_(1,35)_ = 24.333, *p* < 0.001, *η*^2^ = 0.013, and accuracy was higher, *F*_(1,35)_ = 7.971, *p* = 0.008, *η*^2^ = 0.008, for Congruent than Incongruent trials (see Table 4).

**Table 4:** Stroop response times (seconds) and accuracy means (SEM) for each Exercise and Time (*n* = 36).

		Run				Walk			
		Congruent		Incongruent		Congruent		Incongruent	
Response time (s)	10 Min pre-exercise	1.16	(0.03)	1.17	(0.03)	1.14	(0.03)	1.16	(0.03)
	30 Min	1.10	(0.03)	1.12	(0.03)	1.13	(0.03)	1.17	(0.03)
	60 Min	1.10	(0.03)	1.11	(0.03)	1.11	(0.03)	1.13	(0.03)
	90 Min	1.07	(0.03)	1.10	(0.03)	1.11	(0.03)	1.12	(0.03)
	30 Min post-exercise	1.13	(0.03)	1.15	(0.03)	1.18	(0.03)	1.20	(0.03)
Accuracy	10 Min pre-exercise	0.98	(0.01)	0.98	(0.01)	0.97	(0.01)	0.98	(0.00)
	30 Min	0.98	(0.01)	0.99	(0.00)	0.98	(0.01)	0.98	(0.01)
	60 Min	0.98	(0.01)	0.99	(0.00)	0.98	(0.01)	0.98	(0.01)
	90 Min	0.99	(0.01)	0.99	(0.01)	0.97	(0.01)	0.99	(0.01)
	30 Min post-exercise	0.99	(0.01)	0.98	(0.01)	0.98	(0.01)	0.97	(0.01)

We hypothesized that Stroop performance would be higher during the first hour of the Run than Walk, and then the trend would reverse. In partial support of our hypothesis, analysis of response times revealed an Intensity by Time interaction, *F*_(4,140)_ = 3.867, *p* = 0.005, *η*^2^ = 0.022, in which response times were faster during the Run than during the Walk during 30 and 90 min exercise and after exercise, with all *p*-values < 0.05, but did not differ before exercise or during 60 min exercise, with all *p*-values > 0.23. However, we found no two- or three-way interactions involving Congruency and Intensity for response time, accuracy, or Stroop interference (all *p*-values > 0.26).

##### fNIRS Results

**Figure 3:**
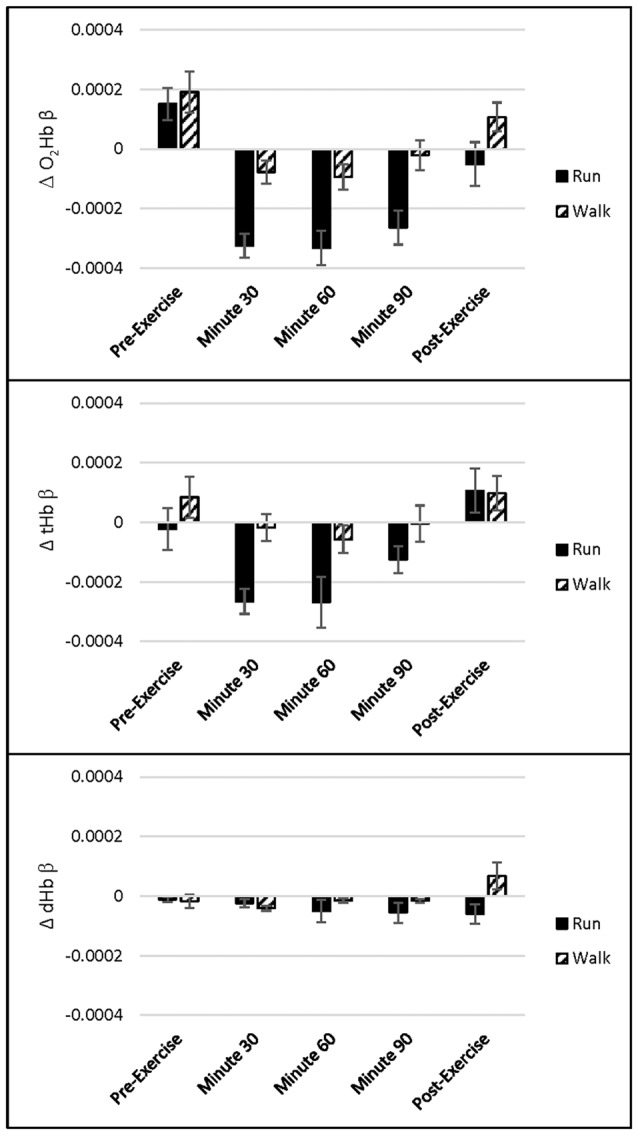
Channel 12 oxygenated hemoglobin (O_2_Hb), total hemoglobin (tHb) and deoxygenated hemoglobin (dHb) concentration (mean ± standard error of the mean (SEM), in μM) for each Exercise and Time during the Stroop test (*n* = 36).

We hypothesized that Stroop-evoked O_2_Hb would increase more during the Run than the Walk during the first hour of exercise, after which point O_2_Hb would decrease during the Run and increase during the Walk. A main effect of exercise Intensity showed that O_2_Hb was lower in response to the Stroop test during the Run than Walk across channels 4, 9, 12 and 18 (*p*s ≤ 0.001). A main effect of Time showed that O_2_Hb was lower in response to the Stroop test during 30, 60 and 90 min exercise than before or after exercise across channels 4, 6–14, 16–17 and 20 (*p*s < 0.001). No effects of Congruency, Intensity by Time interaction, other interactions, or in any other channels were found (*p*s > 0.003) Similarly, for tHb, a main effect of exercise Intensity showed that tHb was lower in response to the Stroop test during the Run than Walk across channels 12 and 18 (*p*s ≤ 0.002). A main effect of Time showed that tHb was lower in response to the Stroop test during 30, 60 and 90 min exercise than before or after exercise across channels 4, 6–8, 12–13 and 16 (*p*s ≤ 0.002). No effects of Congruency, Intensity by Time interaction, other interactions, or in any other channels were found (*p*s > 0.005). For dHb, a main effect of Time showed that dHb was lower in response to the Stroop during 30 and 60 min exercise than before, after, or during 90 min of exercise across channel 4 (*p* < 0.001). No other significant differences were found for dHb (*p*s > 0.009, see Figure 3 for O_2_Hb, dHb and tHb representative channel 12).

#### Does Endurance Exercise Influence the Cognitive Control of Emotion and Associated Changes in PFC Oxygenation on the Cognitive Reappraisal Task?

##### Behavioral Results

Analysis of ratings of unpleasant emotion suggested that the pictures induced negative emotion as intended and that the sample exhibited successful reappraisal, in that ratings of negative emotion were higher for Negative/Maintain trials (mean ± standard error of the means (SEM) = 5.16 ± 0.14) than for Negative/Decrease trials (mean ± SEM = 4.80 ± 0.14), which in turn was higher than for Neutral/Maintain trials (mean ± SEM = 1.32 ± 0.04), *F*_(2,70)_ = 556.09, *p* < 0.001, *η*^2^ = 0.919. We hypothesized that cognitive reappraisal success would be lower after Running than Walking. However, reappraisal success did not differ between the Run (mean ± SEM = 0.45 ± 0.14) and Walk (mean ± SEM = 0.28 ± 0.13; *p* = 0.141).

Descriptive statistics from the open-ended portion of the post-experiment questionnaire are presented in Table 5. Repeated measures ANOVAs with Intensity (Run, Walk) as the within-participants variable indicated that a higher proportion of participants used other emotion regulation strategies, *F*_(1,35)_ = 5.339, *p* = 0.027, *η*^2^ = 0.132, and multiple emotion regulation strategies, *F*_(1,35)_ = 5.000, *p* = 0.032, *η*^2^ = 0.125, following the Walk than following the Run, but use of cognitive reappraisal did not differ as a function of exercise intensity (*p* = 0.600).

**Table 5:** Uninstructed emotion regulation strategies within the cognitive reappraisal task means (SEM) for each Exercise (*n* = 36).

	Overall		Run		Walk	
CR present	0.81	(0.04)	0.83	(0.06)	0.78	(0.07)
Non-CR present	0.29	(0.05)	0.17	(0.06)	0.42	(0.08)
Multiple strategies	0.11	(0.04)	0.03	(0.03)	0.19	(0.07)

**Figure 4:**
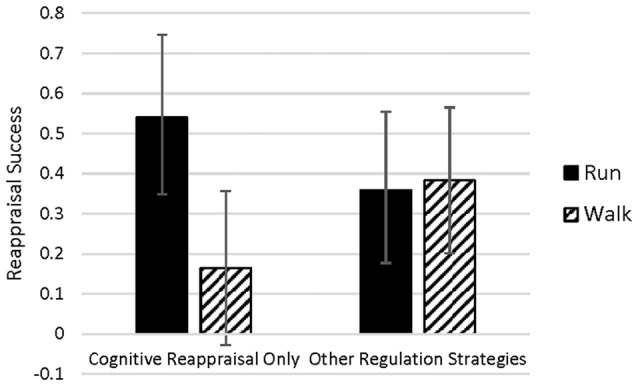
Reappraisal success means (SEM) within participants who adhered to the cognitive reappraisal instruction only (*n* = 17) and those who utilized other uninstructed emotion regulation strategies (*n* = 19) after each Exercise.

To determine whether participants’ use of uninstructed emotion regulation strategies influenced the extent to which they rated the pictures as unpleasant, analysis of reappraisal success was repeated using only participants who employed cognitive reappraisal as the sole emotion regulation strategy (*n* = 17). As shown in Figure 4, reappraisal success was significantly greater following the Run than following the Walk in participants who utilized only cognitive reappraisal, *t*_(16)_ = 2.267, *p* = 0.038, Cohen’s *d* = 0.550, but did not differ between the Run and Walk in participants who utilized other emotion regulation strategies (*n* = 19; *p* = 0.862).

##### fNIRS Results

**Figure 5:**
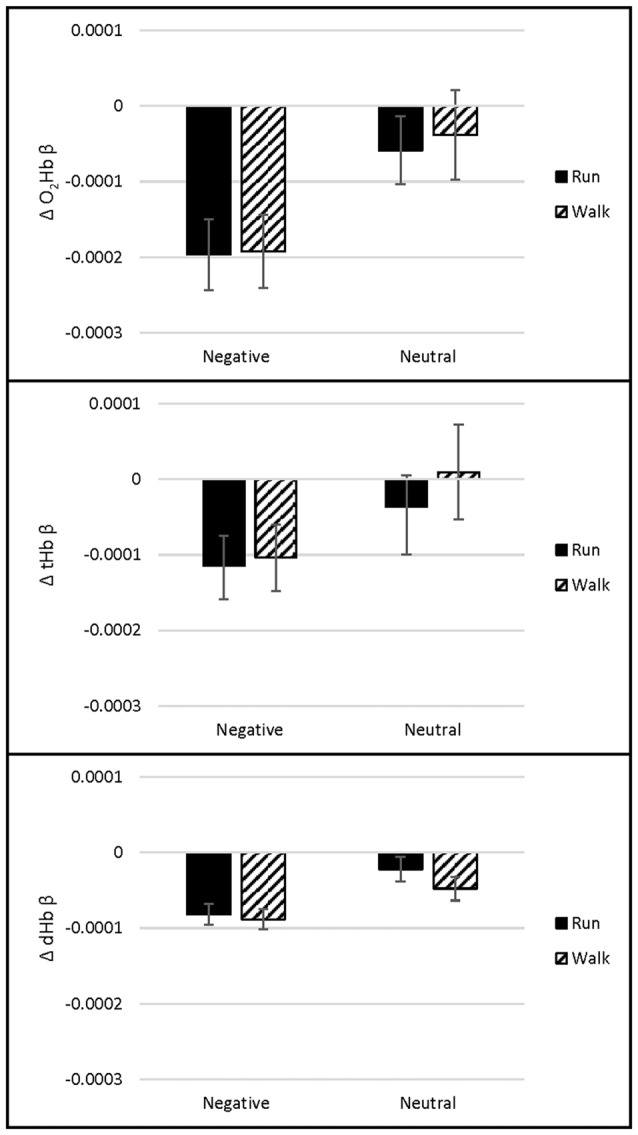
Channel 4 O_2_Hb, tHb and dHb β weight means (SEM) during the CRT preparatory period (upon picture presentation *before* decrease/maintain instruction; *n* = 36).

Cortical activation during the CRT was analyzed separately for the: (1) “preparatory period” consisting of the first 4 s of each trial in which participants viewed the Negative and Neutral pictures, i.e., *before* participants heard the instruction to decrease or maintain; and (2) “regulation period” consisting of the next 8 s of each trial *upon* hearing the instruction to decrease or maintain. During the preparatory period, a main effect of picture Valence showed that O_2_Hb was lower in response to Negative than Neutral pictures across channel 3 (*p* = 0.001). No effect of exercise Intensity, Intensity by Time interaction, or in any other channel were found (*p*s > 0.01). For dHb, a main effect of picture Valence showed that dHb was lower in response to Negative than Neutral pictures across channel 4 (*p* = 0.001). No effect of exercise Intensity, Intensity by Time interaction, or in any other channel were found (*p*s > 0.01). No main effects or interactions were found for tHb in any channel (*p*s > 0.014, see Figure 5 for O_2_Hb, dHb and tHb representative channel 4).

**Figure 6:**
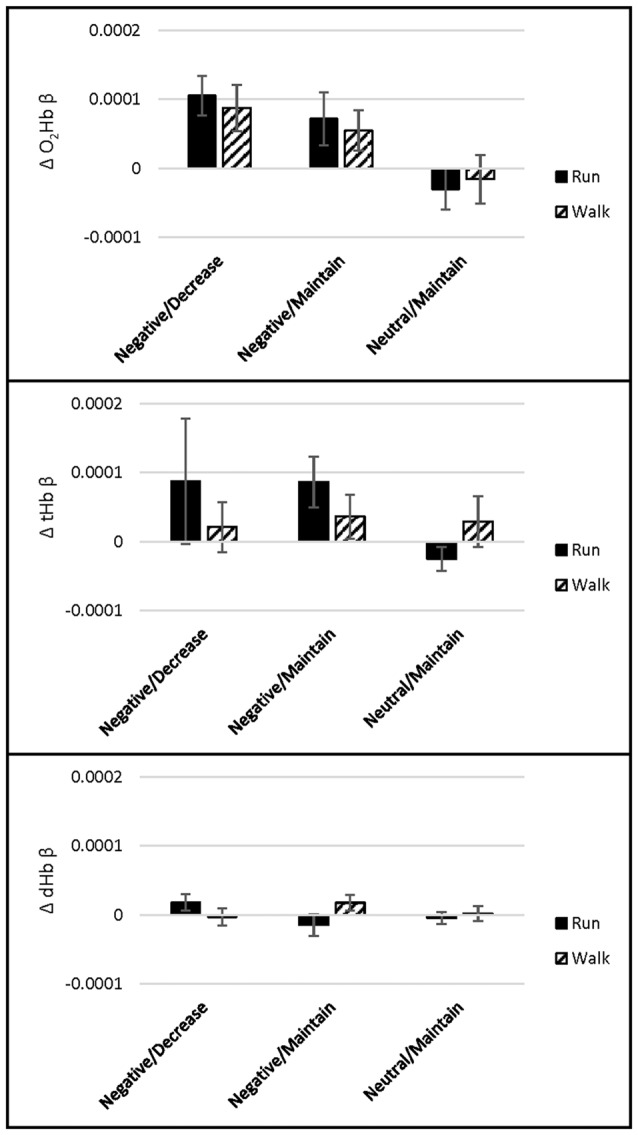
Channel 4 O_2_Hb, tHb and dHb β weight means (SEM) during the CRT regulation period (*upon* decrease/maintain instruction; *n* = 36).

During the regulation period, a main effect of exercise picture Valence/Instruction showed that O_2_Hb was higher in response to Negative pictures, both when instructed to Decrease and Maintain, than Neutral pictures across channels 4 and 16 (*p*s ≤ 0.001). No effect of exercise Intensity, Intensity by Time interaction, or in any other channel were found (*p*s > 0.003). For tHb, a main effect of exercise picture Valence/Instruction showed that tHb was higher in response to Negative pictures, both when instructed to Decrease and Maintain, than Neutral pictures across channels 2 and 4 (*p*s = 0.001). No effect of exercise Intensity, Intensity by Time interaction, or in any other channel were found (*p*s > 0.006). No main effects or interactions were found for dHb (*p*s > 0.008, Figure 6 for O_2_Hb, dHb and tHb representative channel 4).

## Discussion

The present experiment evaluated the influence of endurance exercise (i.e., Run relative to Walk) on emotion, domain-general cognitive control, the cognitive control of emotion, and associated changes in PFC oxygenation. Endurance exercise improved response times and reduced Stroop test-evoked PFC O_2_Hb relative to the Walk, with no concurrent change in selective attention. Further, the Run enhanced cognitive reappraisal adherence and success.

### Endurance Exercise and Emotion

Participants completed measures of emotional state before, every 30 min during, and after the 90-min Run or Walk. Participants generally felt positive throughout both the Run and Walk and the Run augmented positive emotion and arousal more so than the Walk (see Table 3). Such results support the dual-mode theory (Ekkekakis, 2003), which posits that at low to moderate intensities, cognitive factors such as exercise self-efficacy and appraisals of goals determine affective responses, which are generally positive.

### Endurance Exercise and Domain-General Cognitive Control

Participants also completed the Stroop test of selective attention before, every 30 min during, and after the 90-min Run or Walk. Participants exhibited shorter response times during the run than during the walk (see Table 4). Such speeded response times during exercise support the first premise of Dietrich’s RAH model of acute exercise, in that exercise facilitated efficiency of peripheral motor processes (Dietrich and Audiffren, 2011).

Stroop interference did not differ between the two exercise conditions. Thus, we did not support the second premise of the RAH model, in that exercise did not impair higher-order cognitive function. Despite the prevalence of the RAH model, there is little consensus on if and how exercise influences non-motor cognitive processes. While a number of studies have found that exercise influences cognitive control (e.g., Dietrich and Sparling, 2004; Davranche and McMorris, 2009; Del Giorno et al., 2010), others had findings similar to those of the present study, i.e., improved response times with no concurrent deterioration in cognitive control (Kamijo et al., 2007; Davranche et al., 2009; Tempest et al., 2017).

PFC O_2_Hb and tHb increased in response to the Stroop test before exercise and decreased during exercise, while dHb did not change (see Figure 3). Such task-evoked reductions in O_2_Hb and tHb were larger during the Run than Walk but were generally stable within the 90-min Run and Walk. Task-evoked reductions were restricted to a subset of channels, spatially distributed across the PFC. Task-evoked reductions were also relative to the 30–60 s before each iteration of the task. The choice to reference changes within each exercise time point enables us to detect changes that occur during cognitive control, relative to no cognitive control, during exercise. However, it does not allow us to identify changes during exercise, relative to no exercise. Thus future research should employ a consistent, pre-exercise baseline period, to determine both exercise- and cognitive control-evoked changes to PFC oxygenation. Recent research suggests that cognitive control tasks increased O_2_Hb during 60 min very low-intensity exercise, but did not influence O_2_Hb during heavy-intensity exercise, akin to 10% above the ventilatory threshold (Tempest et al., 2017), perhaps due to extra-cerebral responses to vigorous intensities, such as surface blood flow (discussed below), respiration, and movement. Thus, it is possible that in both studies, extra-cerebral responses masked cerebral ones. As Tempest et al. (2017) suggest, future work should control for these extra-cerebral responses. Future work should also utilize new, wearable magnetoencephalography (MEG), which provides higher spatiotemporal resolution than NIRS (Boto et al., 2018).

### Endurance Exercise and the Cognitive Control of Emotion

Participants completed the CRT following their 90-min run and walk, which involved cognitively reappraising and maintaining their emotional responses toward negative and neutral pictures. Participants better adhered to the instructed emotion regulation strategies following the run, as they tended to use more uninstructed strategies following the walk (see Table 5). Within participants who adhered to the cognitive reappraisal instruction, running improved reappraisal success relative to the walk (see Figure 4). Thus, running enhanced the extent to which participants utilized cognitive reappraisal, and the success with which they used it. These results are in line with previous research showing that habitual exercise is associated with enhanced cognitive reappraisal success (Giles et al., 2017), and that acute exercise ameliorates the impact of emotion regulation difficulties on negative emotional responses (Bernstein and McNally, 2017). Approximately half of participants in the present experiment employed only cognitive reappraisal, and thus future research should look to replicate the effect in larger samples.

Changes in PFC oxygenation during the CRT were limited to those driven by picture valence, with no effects of exercise intensity or emotion regulation instruction (see Figures 5, 6). During the preparatory period (i.e., the 4 s prior to delivery of the regulation instruction), O_2_Hb and dHb were more negative in response to negative than to neutral pictures, each in one channel. During the regulation period (i.e., the next 8 s during which participants employed the emotion regulation strategies), O_2_Hb and tHb were higher in response to negative than neutral pictures, each in two channels, regardless of whether participants were instructed to reappraise or maintain their emotions. Such results support those from our lab (Giles et al., 2017), and others, which found increased O_2_Hb in response to emotional stimuli such as a stress-inducing mental arithmetic task (Tanida et al., 2004), pleasant and unpleasant film clips (Leon-Carrion et al., 2006) and unpleasant pictures (Herrmann et al., 2003). However, the direction of PFC response to emotion is a topic of continued inquiry, as it may depend on task demands (Herrmann et al., 2003) and individual differences (Hoshi et al., 2011). The absence of differences between exercise intensities may be attributed to: (a) quick return to baseline of PFC oxygenation following exercise (Fumoto et al., 2010); or (b) similarity between two exercise intensities, discussed below.

### Limitations

The present findings suggest that moderate-intensity endurance exercise enhances positive emotion and emotion regulation success using cognitive reappraisal. We did not find evidence of endurance exercise influence on cognitive control. However, four primary limitations curtail our ability to generalize the findings to exercise as a whole. First, participants’ average exertion ratings of “somewhat hard” during the run suggest that despite the relatively long duration, they did not reach an intensity akin to the ventilatory threshold or respiratory compensation threshold at which emotional responses and cognitive control would decline. Given that changes in emotion and cognitive control may occur more as a function of exercise intensity than duration (Kilpatrick et al., 2007), future research should increase the range of exercise intensities to better understand thresholds at which domain general cognitive control declines may emerge. Similarly, we did not include a sedentary group of individuals, for comparison of physical fitness. Given that less fit individuals experience exercise as more negative and fatiguing than more highly fit individuals (Ekkekakis et al., 2011), future research should examine changes in emotion and cognitive control during exercise in sedentary individuals.

Second, participants in the present study were free from psychological disorders. However, depression, anxiety and stress influence cognitive control and the cognitive control of emotions (Castaneda et al., 2008). Thus, future research should examine how individual differences in these mood patterns relate to exercise-associated changes in cognitive control and emotion.

Third, the Stroop test in its present iteration was perhaps not sensitive to changes that may occur as a function of exercise. Previous studies have utilized more challenging versions of the Stroop test (Yanagisawa et al., 2010; Lucas et al., 2012; Endo et al., 2013) and other tasks of cognitive control, such as the Wisconsin Card Sorting Task (Dietrich and Sparling, 2004) and Eriksen flanker task (Ando et al., 2011). Further, the Stroop test, and similar tests using contrast measures, are subject to low reliability due to error variance being additive (Strauss et al., 2005; White et al., 2018). Thus, it is currently unclear whether a 90-min bout of moderate-intensity exercise influences cognitive control, or whether our failure to support the RAH model (Dietrich and Audiffren, 2011) is a function of the specific task implemented.

Finally, near-surface blood flow (e.g., skin blood flow) has been shown to increase during exercise, and changes to skin blood flow may correlate with changes in cerebral O_2_Hb (Miyazawa et al., 2013). Thus, it is difficult to disentangle increases in surface blood flow from cortical blood flow occurring as a result of exercise. Short source-detector separation optodes have been employed to account for superficial/systemic interference (Gagnon et al., 2012). Such a short source-detector channel was not included in the present optode array, and should be considered for future exercise fNIRS work. Nevertheless, given that skin blood flow generally increases during exercise, and we witnessed reductions in PFC oxygenation, skin blood flow is unlikely to entirely account for changes in PFC oxygenation.

## Conclusion

The present experiment suggests that endurance exercise akin to 90 min moderate-intensity running increases positive emotion during exercise, and the cognitive control of emotion using reappraisal after exercise. Further, although endurance exercise reduced PFC activation, domain-general cognitive control remained stable. Thus, even at relatively moderate intensities, endurance athletes benefit emotionally from running both during and after exercise, and task-related PFC oxygenation reductions do not appear to hinder prefrontal-dependent cognitive control. However, it remains unclear whether the same effects would persist at higher exercise intensities, akin to athletes’ high intensity training and race experience, or in response to more challenging cognitive control tasks. Thus, the findings add to the growing body of literature demonstrating complex relationships between endurance exercise, emotion, and emotion regulation, and future research should expand upon the conditions under which endurance exercise may enhance or impair emotion and cognitive control.

## Data Availability

The datasets for this manuscript are not publicly available because the funding agency does not permit public release of data products. Requests to access the datasets should be directed to Grace Giles at grace.giles@tufts.edu.

## Author Contributions

GG, ME, TB, HU, CM, HT and RK contributed to study concept. GG completed data preparation and wrote the first draft of the manuscript. GG, MD, TB, HU, HG and RB contributed to data analysis. All authors contributed to manuscript revision, read and approved the submitted version.

## Conflict of Interest Statement

Drs. Barbour and Graber are affiliated with Photon Migration Technologies, Corp, which is the parent company of the manufacturer, NIRx Medical Technologies, LLC, of the NIRS device used in this study. The remaining authors declare that the research was conducted in the absence of any commercial or financial relationships that could be construed as a potential conflict of interest.
