# Supplementary material for: Endurance Exercise Enhances Emotional Valence and Emotion Regulation
Source: Front Hum Neurosci. 2018 Oct 16;12:398. doi: 10.3389/fnhum.2018.00398 (PMC6232759; doi:10.3389/fnhum.2018.00398)
Supplement: Supplementary file 1 [file Table_1.docx]

**Appendix A. Questionnaire descriptions**

Internal consistency assessments (α coefficients), where appropriate, are based on responses from study participants.

*Godin Leisure Time Questionnaire:* The Godin Leisure Time Exercise Questionnaire was used to quantify participants’ activity level, and asks participants the number of times they engage in strenuous, moderate, and light exercise for at least 15 minutes over an average week (Godin and Shephard, 1985). A weekly leisure activity score was calculated by multiplying the number of times participants engaged in strenuous, moderate and light exercise for at least 15 minutes by 9, 5, and 3, respectively, and summing the resulting scores. Individuals who score at least 24 are considered active and those who score less than 14 are considered inactive (Godin, 2011).

*Beck Depression Inventory (BDI):* The BDI is a scale commonly used to quantify depressive symptoms (Beck et al., 1961). It contains 21 self-report statements in which participants rate attitudes and thoughts associated with depression, such as *sadness, pessimism, loss of interest, changes in appetite,* and *fatigue* on scales ranging from 0 to 3, e.g. *Sadness*: 0 = I do not feel sad, 1 = I feel sad much of the time, 2 = I am sad all the time, 3 = I am so sad or unhappy that I can’t stand it and *Pessimism*: 0 = I am not discouraged about my future, 1 = I feel more discouraged about my future than I used to be, 2 = I do not expect things to work out for me, 3 = I feel my future is hopeless and will only get worse. Item scores were summed to create a composite score, which ranged from 0 to 63 (α = .66). Scores of less than 10 indicate minimal depression, 10-18 indicate mild to moderate depression, 19-29 indicate moderate to severe depression, and 30-63 indicate severe depression (Beck et al., 1988).

*Perceived Stress Scale (PSS):* The PSS is used to quantify situations that individuals appraise as stressful in their life (Cohen et al., 1983). It consists of 14 questions assessing how often participants experienced a number of stress-related factors in the past month, such as loss of control (e.g. *In the last month, how often have you been upset because of something that happened unexpectedly?*), feeling nervous or stressed (e.g. *In the last month, how often have you felt nervous and "stressed"*?), and ability to cope (e.g. *In the last month, how often have you felt that you were on top of things?*), among others. Items were measured on a five-point scale ranging from “never” (0) to “very often” (4). Items assessing the absence of stress were reverse scored, and item scores were then summed. Scores ranged from 0 to 40, with higher scores indicating higher perceived stress (α = .85).

*Emotion Regulation Questionnaire (ERQ):* The ERQ measures individual differences in the habitual use of two emotion regulation strategies: cognitive reappraisal and expressive suppression (Gross and John, 2003). Participants were asked to respond to statements on a seven-point scale ranging from “strongly disagree” (1) to “strongly agree” (7) to statements concerning reappraisal, e.g. *When I want to feel less negative emotion, I change the way I’m thinking about a situation*, and expressive suppression, e.g. *I control my emotions by not expressing them*. Item scores for the cognitive reappraisal and expressive suppression scales were averaged, and scores ranged from 1-7, with higher scores indicating greater reliance on cognitive reappraisal (α = .659) and expressive suppression (α = .83).

*State–Trait Anxiety Inventory - Trait (STAI-T)*: The STAI-T is composed 10 items that measure trait anxiety (Spielberger et al., 1983). Participants were asked to indicate the emotions they “generally” feel ranging from “almost never” (1) to “almost always” (4), such as *I am inclined to take things hard, I am a steady person,* and *I feel blue*. Items assessing the absence of anxiety were reverse scored, and item scores were then summed. Scores ranged from 20-80 with higher scores indicating greater anxiety (α = .75).

**Appendix B. Heart rate and perceived exertion results**

**Heart Rate (HR)**

To assess exercise- and time-dependent changes in heart rate, heart rate was averaged into 100 1-minute time intervals (i.e. 5 minute warmup, 90 minute Run/Walk, 5 minute cooldown), which were then analyzed using an ANOVA with exercise Intensity (Walk, Run) and Time (Minutes 0, 2,…, 99) as within- participant factors using the Bonferroni correction to correct for multiple comparisons. Thus p = 0.05 divided by 100 minutes resulted in p = 0.0005.

HR analyses were based on 35 participants, as the monitor failed to record data for one participant. Main effects for Time, *F*(98,3332) = 153.49, *p* < 0.001, η^2^ = 0.124, Intensity, *F*(1,34) = 390.001, *p* < 0.001, η^2^ = 0.720, and an Intensity-by-Time interaction, *F*(98, 3332) = 75.287, *p* < 0.001, η^2^ = 0.048, were found. Such effects were characterized by similar heart rates during the warm-up (i.e. Minutes 0-4; all *p-*values > 0.03) and higher heart rate in the Run than Walk condition when participants began their 90-minute Run or Walk (i.e. Minutes 5-99; all *p-*values < 0.001). Such results confirm that participants reached and maintained their target heart rates, and that heart rate was higher during and after the Run than Walk.

Heart rate (HR) means (SEM) in beats per minute (BPM) for each Exercise and Time during the warm-up (minutes 0 - 4), walk or run (minutes 5 - 94), and cool-down (minutes 95-99) (n=35).

**Rated Perceived Exertion Scale (RPE**)

Analysis of the Perceived Rated Exertion (RPE) showed that rated exertion was higher in the Run than Walk condition, *F*(1,35) = 22.599, *p* < 0.001, η^2^ = 0.273. No effect of time or intensity by interaction were found (all *p-*values > 0.56). Such results confirm that participants perceived greater exertion levels during the Run than Walk.

Rated Perceived Exertion Scale (RPE) means, standard error of the means (SEM), medians, and interquartile ranges (ICQ) for each Exercise and Time (n=36).

|  |  | Mean | SEM | Median | IQR |
| --- | --- | --- | --- | --- | --- |
| Min 30 | Run | 11.4 | 0.3 | 12.0 | 2 |
|  | Walk | 9.7 | 0.3 | 10.0 | 3 |
| Min 60 | Run | 12.3 | 0.2 | 12.0 | 1 |
|  | Walk | 10.3 | 0.3 | 10.5 | 3 |
| Min 90 | Run | 13.0 | 0.3 | 13.0 | 2 |
|  | Walk | 10.4 | 0.3 | 11.0 | 3 |

**Appendix C. Trial structure of the Cognitive Reappraisal Task**

+


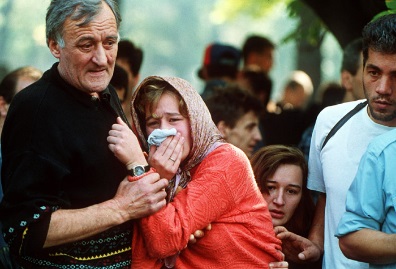
**

**

👂

“decrease”

or

“maintain”

•

******

Trial structure of the Cognitive Reappraisal Task. Photo of mourning family from Evstafiev (1992) (Urry, 2010). Used with permission of the photographer.

**Appendix D. Stroop Test Supplementary Results**

Stroop test response time and accuracy were analyzed using repeated measures ANOVA with Intensity (Run, Walk), Time (Pre-Exercise, Post-Exercise and, separately, Minute 30, Minute 60, Minute 90), and Congruency (Congruent, Incongruent) as fixed factors. Stroop interference (Incongruent – Congruent response time) was analyzed using repeated measures ANOVA with Intensity (Run, Walk) and Time (Pre-Exercise, Post-Exercise and, separately, Minute 30, Minute 60, Minute 90) as fixed factors.

In analyses comparing Stroop data before versus after exercise, response times were shorter, *F*(1,35) = 11.43, *p* = 0.002, η^2^ = 0.011, and accuracy was higher, *F*(1,35) = 8.363, *p* = 0.007, η^2^ = 0.015, for Congruent than Incongruent trials. Analysis of response times revealed an Intensity by Time interaction, *F*(1, 35) = 8.420, *p* = 0.006, η^2^ = 0.053, in which response times were shorter after the Run than after the Walk (*p* = .004), but did not differ before exercise (*p* = .397). However, we found no two- or three-way interactions involving Congruency and Intensity for response time or accuracy (all *p*-values > 0.13). We found no effects for Stroop interference (all *p*-values > 0.17).

During exercise, response times were shorter, *F*(1,35) = 20.989, *p* < .001, η^2^ = 0.022, and accuracy was higher, *F*(1,35) = 5.007, *p* = 0.032, η^2^ = 0.006, for Congruent than Incongruent trials. Response times were shorter in the Run than Walk, *F*(1, 35) = 5.081, *p* = .031, η^2^ = 0.061 and during 90 minutes exercise than 30 and 60 minutes exercise, *F*(2, 70) = 7.794, *p* = .001, η^2^ = 0.039. We hypothesized that Stroop performance would be higher during the first hour of the Run than Walk, and then the trend would reverse. In partial support of our hypothesis, analysis of response times revealed a Congruency by Intensity by Time interaction, *F*(2, 70) = 3.260, *p* = 0.044, η^2^ = 0.003, in which response times were shorter during the Run than during the Walk during 30 and 90 minutes exercise, with all *p­*-values < 0.05, but did not differ during 60 minutes exercise (*p* = .231). Response times were also shorter for Congruent than Incongruent trials during 30- and 90-minutes exercise, with all *p­*-values < 0.01, but did not differ during 60 minutes exercise (*p* = .063). However, we found no Congruency x Exercise interaction at any time point during exercise. For Stroop interference, we found an Intensity by Time interaction, *F*(2, 70) = 3.260, *p* = 0.044, η^2^ = 0.031, but follow-up tests were not significant (all *p*-values > 0.10).
